# Dietary biomarkers: advances, limitations and future directions

**DOI:** 10.1186/1475-2891-11-109

**Published:** 2012-12-14

**Authors:** Valisa E Hedrick, Andrea M Dietrich, Paul A Estabrooks, Jyoti Savla, Elena Serrano, Brenda M Davy

**Affiliations:** 1 Department of Human Nutrition, Foods and Exercise, 221 Wallace Hall (0430), Virginia Tech, Blacksburg, Virginia, 24061, US; 2 Department of Civil and Environmental Engineering, Virginia Tech, Blacksburg, Virginia, 24061, US; 3 Department of Human Development and Center for Gerontology, Virginia Tech, Blacksburg, Virginia, 24061, US

**Keywords:** Dietary biomarkers, Dietary assessment, Metabolomics

## Abstract

The subjective nature of self-reported dietary intake assessment methods presents numerous challenges to obtaining accurate dietary intake and nutritional status. This limitation can be overcome by the use of dietary biomarkers, which are able to objectively assess dietary consumption (or exposure) without the bias of self-reported dietary intake errors. The need for dietary biomarkers was addressed by the Institute of Medicine, who recognized the lack of nutritional biomarkers as a knowledge gap requiring future research. The purpose of this article is to review existing literature on currently available dietary biomarkers, including novel biomarkers of specific foods and dietary components, and assess the validity, reliability and sensitivity of the markers. This review revealed several biomarkers in need of additional validation research; research is also needed to produce sensitive, specific, cost-effective and noninvasive dietary biomarkers. The emerging field of metabolomics may help to advance the development of food/nutrient biomarkers, yet advances in food metabolome databases are needed. The availability of biomarkers that estimate intake of specific foods and dietary components could greatly enhance nutritional research targeting compliance to national recommendations as well as direct associations with disease outcomes. More research is necessary to refine existing biomarkers by accounting for confounding factors, to establish new indicators of specific food intake, and to develop techniques that are cost-effective, noninvasive, rapid and accurate measures of nutritional status.

## Introduction

Collecting dietary intake data is associated with many challenges, which are primarily related to the subjective nature of data collection tools such as food frequency questionnaires (FFQ), multiple-day food records and 24-hour dietary recalls. Individuals are not always able to recall all foods consumed or the specific components of the food (e.g., condiments in sandwiches), have difficulty determining accurate portion sizes and typically underreport dietary intake [1-4]. Each method has strengths and limitations; however, food records and dietary recalls typically are costly (resource-intensive), time consuming, place a high burden on respondents, provide only recent intake information (i.e., not habitual intake patterns) and are not always feasible in large-scale investigations or in those including low income or low literacy populations [4-6]. A FFQ may provide a glimpse into a population’s habitual dietary intake over time, whereas food records and dietary recalls assess days/weeks, which may be more precise but not representative of typical intake over time [4]. Additionally, several technological advances in dietary assessment methods have occurred over the past few years, specifically with computer software and web-based applications [7]. The Nutrition Data System for Research, developed by the University of Minnesota, is a commonly utilized software program that allows for interview-administered 24 hour dietary recalls as well as researcher-entered multiple day food records [8]. The automated multiple-pass method is a 24-hour dietary intake assessment method that is automated but still interview administered [7]. Most recently, the automated self-administered 24-hour dietary recall (ASA24) has been developed and based off of the automated multiple-pass method. In addition to the ASA24 being self-administered, further benefits include the format of a free web-based tool that can be accessed with internet at any location; thus decreasing researcher and participant burden [9]. Although these advances are promising, as well as cost-effective, using self-reported dietary intake methods to assess dietary intake is still not without intake error, a commonly cited research limitation [3]. In contrast, biomarkers of food or nutrient intake (or exposure) are able to objectively assess dietary intake/status without the bias of self-reported dietary intake errors [10-12], and also overcome the problem of intra-individual diet variability [2]. The Institute of Medicine has recognized the lack of nutritional biomarkers as a knowledge gap requiring future research, including: 1) the need for biomarkers that can predict functional outcomes and chronic diseases, and 2) the need to improve dietary assessment and planning methods [10]. Dietary biomarkers are not without limitations; cost and degree of invasiveness are factors to consider [3]. Therefore, the need for non-invasive, inexpensive and specific dietary markers is clear [10].

Dietary biomarkers are desirable for their ability to more accurately assess nutritional intake/status versus self-reported methods, validate self-reported intake measures, evaluate intake of dietary items when food-composition databases are inadequate, and to more accurately associate dietary intake with disease risk and nutritional status [13]. Biomarkers can be categorized into short-term (reflecting intake over past hours/days), medium-term (reflecting intake over weeks/months) and long-term markers (reflecting intake over months/years), with the type of sample used being a main determinant of time (e.g., blood, hair, adipose tissue) [13].

Although dietary biomarkers generally provide a more proximal measure of dietary intake, factors which may not present in traditional dietary assessment methods could skew biomarker measures of dietary intake. Such factors could include genetic variability, lifestyle/physiologic factors (e.g., smoking), dietary factors (e.g., nutrient-nutrient interaction), biological sample and analytical methodology [14]. Unfortunately, little research is available which addresses this issue. As a result, it is imperative to assess a biomarker’s validity, reproducibility, ability to detect changes over time and robustness across diverse populations, as well as strengths and limitations to ensure it is evaluated using the proper techniques.

As the profession of dietetics and health sciences trends towards individualized nutrition [12,15], developing biomarkers that measure intake of specific foods, rather than nutrients, may become a primary focus [2]. The emerging field of metabolomics in human nutrition may advance the discovery of novel biomarkers for specific dietary intake and consequently health status [16]. Metabolomics is the identification of small molecule metabolites and nutrients available in biological fluids (blood, saliva, urine, etc.) that makes up the metabolome [17,18]. The metabolites are the products of metabolism of medicines, foods, toxins, etc. [18,19]. Metabolomics has been used to identify dietary intake patterns by identifying the molecules that vary between different diets [17], which can be useful in determining potential markers of diet-disease risk [20], as well as the potential to discover novel biomarkers for specific foods [21]. The availability of biomarkers that provide estimates of specific foods and dietary components intake could greatly enhance nutritional research targeting compliance to national recommendations, such as the U.S. 2010 Dietary Guidelines and those of the American Heart Association. The purpose of this review is to present and evaluate available literature regarding the validity, reliability and sensitivity of current dietary biomarkers for macronutrient dietary component/foods (carbohydrates, fats, proteins), as well as food/nutrients which cannot be categorized within macronutrients (e.g., caffeine). To our knowledge, no review has addressed biomarkers for intake of specific foods and dietary components. Therefore the present review will include an evaluation of research investigating novel biomarkers for specific foods/dietary components (e.g., ^13^C for corn and cane sugars [22]). This review aims to provide a critical examination of the available methods for measuring traditional macronutrient intake/status that have been updated or modified in the past decade and assess validity, reproducibility and sensitivity of proposed and accepted biomarkers.

## Methods

**Figure 1:**
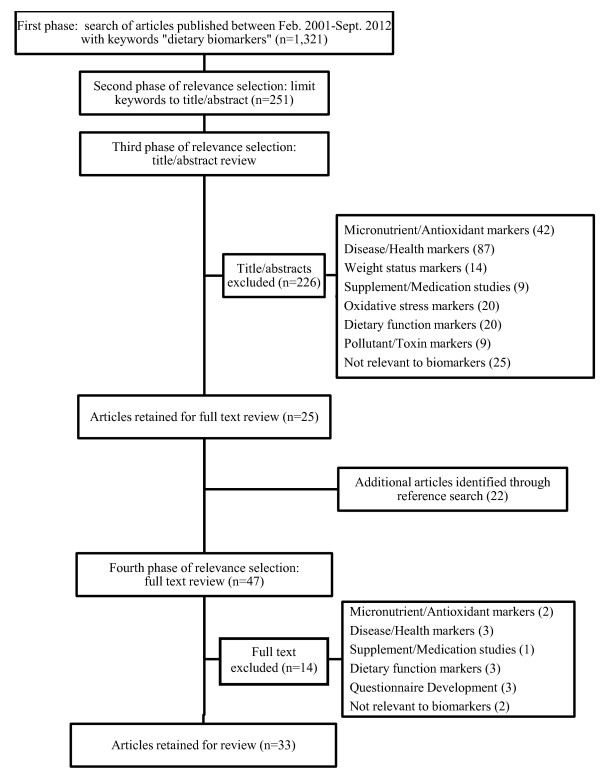
Flow diagram of the structured review of dietary biomarkers.

A literature search (Figure 1) was conducted in September 2012. Stage 1 consisted of an electronic search of the keywords “dietary biomarkers” using PubMed (MEDLINE database). The review was limited to clinical trials, meta-analysis, randomized controlled trials, validation studies, journal articles and reviews published within the past decade (Feb. 2001-Sept. 2012). This search identified 1,321 articles, which was further refined by limiting the search to title/abstract text (n=251). Stage 2 involved a review of article title and abstract, which identified 118 articles. At Stage 3, full texts of papers were downloaded and assessed further for exclusion/inclusion criteria. To be included in the review, the focus of the article had to be intake biomarkers of macronutrients or specific foods/dietary component intake. Exclusion criteria included the following: biomarkers of disease-risk/health status (e.g. cancer markers); biomarkers associated with weight status, dietary supplements or medicines; biomarkers of oxidative stress; micronutrient or antioxidant biomarkers; pollutant or toxin biomarkers; and biomarkers of dietary item function rather than biomarker of intake (e.g., effect of fiber on colon health). Figure 1 presents an overview of the review process.

## Results

Thirty-three articles were identified for inclusion. Biomarkers were categorized under their respective macronutrient, as well as an additional category for specific dietary components that did not fall within the macronutrient category (e.g., caffeine). Research findings are summarized in the text in the following order: macronutrients (carbohydrates, fats and proteins) and specific foods/dietary components. Recent literature related to biomarkers for dietary macronutrients (carbohydrate, fat and protein containing foods) is summarized in Table 1.

**Table 1:** Summary of recent biomarker studies related to macronutrient foods

**Food/Dietary component**^**a**^	**Reference**	**Biomarker**^**b**^**(Sample size)**	**Biological sample**	**Analytic procedure**^**c**^	**Biomarker class**^**d**^	**Validity**^**e,f**^**(p value)**	**Reproductivity**^**f**^**(p value)**	**Sensitivity**^**g**^
*Carbohydrates*								
Cane Sugar/HFCS	Cook et al. (2009)	^13^C in blood glucose (5 young adults)	Plasma	GC-IRMS	Short term	R^2^=0.90 (<0.001)		
	Yeung et al. (2010)	^13^C (186 older adults)	Serum (fasting)	CF-SIRMS		r=0.87 (0.01)		
	Davy et al. (2011)	^13^C (60 adults)	Fingerstick	NA-SIMS	Medium term?	r=0.365 (<0.05)	r=0.873 (<0.001)	
Sugar	Tasevska et al. (2005)	Sucrose & Fructose (12 male adults; 13 adults)	Urine (24 hr)	Enzymatic	Short term	R^2^= 0.888 (<0.001)	ICC 0.67^i^	+
	Kuhnle et al. (2008)	Sucrose (7 adults)	Urine (24 hr)	GC-MS	Short term			
			Urine (24 hr)	LC-MS	Short term			
Whole-Grain Wheat/Rye	Linko-Parvinen et al. (2007)	Enterolactone (ENL) (15 adults)	Plasma (fasting)	TR-FIA	Short term			
		Total Alkylreoscinol (AR) concentration	Plasma (fasting)	GC-MS	Short term	R^2^=0.939 (<0.05)		
		Erythrocyte AR	RBC (fasting)	GC-MS	Medium term?	R^2^=0.854 (<0.05)		
	Aurbertin-Leheudre et al. (2008)	Total AR concentration (56 women)	Plasma (fasting)	GC-MS		r=0.406 (0.003)		
		DHBA	Urine (24 hr)	HPLC		r= 0.359 (0.008)		
		DHPPA	Urine (24 hr)	HPLC		r= 0.402 (0.003)		
	Landberg et al. (2008, 2009, 2009)	DHBA, DHPPA (16 adults)	Urine (24 hr)	HPLC	Short term			
		Total AR concentration (30 adults; 17 males with prostate cancer)	Plasma (fasting)	GC-MS	Short term	r= 0.58 (<0.001)	ICC 0.90, 0.88^i^	+
	Aderson et al. (2011)	Total AR concentration (51 adults)	Plasma (fasting)	GC-MS	Medium term	r=0.53 (<0.001)	r=0.38 (P<0.001)	
*Fats*								
Total Fat	King et al. (2006)	PUFA, MUFA, SFA	RBC (fasting)	1D-TLC	Long term?			+
		(66 postmenopausal females)	Plasma (fasting)	1D-TLC				+
Fatty Acids	Baylin et al. (2002)	PUFA (503 older Costa Ricans)	Adipsoe tissue (fasting)	GLC		r=0.58 (<0.001)		
		*Trans-*fatty acids	Adipose tissue (fasting)	GLC		r=0.43 (<0.001)		
	Poppitt et al. (2005)	SFA, MUFA, PUFA	RBC (fasting)	GC				
		(20 male adults)						
	Fuhrman et al. (2006)	Oleic acid (204 female adults)	RBC (fasting)	GC	Medium term	r=0.45; 0.47* (<0.001)		
		Total PUFA	RBC (fasting)	GC	Medium term	r=0.17^h^; 0.39* (<0.001)		
		Total MUFA	RBC (fasting)	GC	Medium term	r=0.40; 0.48*		
						(<0.001)		
		Total SFA	RBC (fasting)	GC		r=0.14^hi^; 0.07*^hi^		
	Thiebaut et al. (2009)	SFA, MUFA, PUFA (1,114 female adults)	Serum (fasting)	GC	Long term	r=0.16–0.29 (<0.0001)		
Essential Fatty Acids (EFA)	Baylin et al. (2005)	Alpha-linolenic acid & Linoleic acid	Adipose tissue	GLC	Long term	r=0.51; 0.52** (<0.05)		
		(200 Costa Rican adults)	Blood (fasting)	GLC	Long term	r=0.38; 0.43** (<0.05)		
			Plasma (fasting)	GLC	Long term	r=0.39; 0.41** (<0.05)		
	Fuhrman et al. (2006)	Linoleic acid (204 female adults)	RBC (fasting)	GC	Medium term	r=0.23; 0.39* (<0.05; <0.001)		
		Alpha-linolenic acid	RBC (fasting)	GC		r=0.14^hi^, 0.07*^hi^		
EPA, DHA	Kuriki et al. (2003)	EPA (15 male, 79 female Japanese dietitians)	Plasma (fasting)	GC		r=0.57; 0.60*** (<0.05; <0.001)		
		DHA	Plasma (fasting)	GC		r=0.57; 0.30** (<0.05; <0.01)		
	Harris et al. (2004)	Omega-3 Index (EPA+DHA) (57 adults)	Plasma (fasting)	GC	Long term	r=0.91(<0.001)		+
			Blood (fasting)	GC	Long term	r=0.91 (<0.001)		+
	O’Brien et al (2009)	^15^N-EPA (496 adult Eskimos)	Blood	CF-IRMS		r=0.47(<0.001)		
		^15^N-DHA	Blood	CF-IRMS		r=0.46 (<0.001)		
	Nash et al. (2009)	^15^N-EPA (144 adult Eskimos)	Hair	CF-IRMS	Medium term	r=0.83 (0.001)		
		^15^N-DHA	Hair	CF-IRMS	Medium term	r=0.84 (<0.001)		
Olive Oil	Micro-Casas et al. (2002)	Tyrosol (7 adults)	Urine (24 hr)	GC-MS	Short term			
		Hydroxytyrosol	Urine (24 hr)	GC-MS	Short term			
*Proteins*								
Protein	Bingham (2003)	Urine Nitrogen (8 adults)	Urine (24 hr)	Kjeldahl	Short term	R^2^=0.99^i^		
Animal Protein	Petzke & Lemke (2009)	^13^C (14 young adult females)	Hair	GC/C/IRMS	Medium term-Long term?	R^2^=0.44 (0.005)		
		^15^N	Hair	GC/C/IRMS	Medium term- Long term?	R^2^=0.17^h^ (0.069)		
	Cross et al. (2011)	Creatinine (17 male adults)	Urine (24 hr)	Kinetic assay	Short term	P_trend_(<0.0001)		+?
		Taurine	Urine (24 hr)	IEC	Short term?	P_trend_(<0.0001)		+?
		1-methylhistidine	Urine (24 hr)	IEC	Short term?	P_trend_(<0.0001)		+
		3-methylhistidine	Urine (24 hr)	IEC	Short term	P_trend_(<0.0001)		+

^a^HFCS, High fructose corn syrup; EPA, Eicosapentaenoic Acid; DHA, Docosahexaenoic Acid.

^b^DHBA, 3,5-Dihydroxybenzoic Acid; DHPPA, 3-(3,5-Dihydroxyphenyl)-Propanoic Acid; PUFA, Poly-Unsaturated Fatty Acid; MUFA, Mono-Unsaturated Fatty Acid; SFA, Saturated Fatty Acid; RBC, Red Blood Cell.

^c^GC-IRMS, gas chromatography isotope ratio mass; CF-SIRMS, Continuous-flow stable isotope ratio mass spectrometry; NA-SIMS, Natural abundance stable isotope mass spectrometry; GC-MS, Gas chromatography-mass spectrometry; LC-MS, Liquid chromatography-mass spectrometry; TR-FIA, Time-resolved fluroimmunoassay; HPLC, High-performance liquid chromatography; 1D-TLC, One-dimensional thin-layer chromatography; GLC, Gas liquid chromatography; GC, Gas chromatography; CF-IRMS, Continuous-flow isotope ratio mass spectrometry; GC/C/IRMS, Gas chromatography/combustion/ isotope ratio mass spectrometry; IEC, Ion-exchange chromatography.

^d^Short term: hours/days; Medium term: weeks/months; Long term: months/years.

^e^Results of comparsion of biomarkers to an appropriate dietary assessment method.

^f^Representative values from the literature.

^g^Biomarker is able to detect changes over time or distinguish high from low consumers; + = sensitivity has been demonstrated.

^h^Correlation not significant.

^i^Significance not reported.

*Values presented are pre, post menopausal, respectively.

**Values presented are alpha-linolenic, linoleic acid, respectively.

***Values presented are male, female, respectively.

### Carbohydrates

The American Heart Association and the U.S. 2010 Dietary Guidelines provide recommendations for added sugar intake, as it is theorized that added sugars in the diet, particularly sugar-sweetened beverages (SSB), have contributed to the rise in obesity prevalence due to research suggesting that they contribute to excessive energy intake [23-25]. Yet, significant evidence is needed to directly link SSB and added sugar intake to obesity and other co-morbidities such as hypertension, diabetes and cardiovascular disease [26-28]. Valid and reliable biomarkers of sugar intake are needed to support existing dietary recommendations. Additionally, the U.S. 2010 Dietary Guidelines suggest one half of grains consumed should be whole grains [29], due to associations with heart health [30,31]. However, the general population may have difficulty distinguishing whole grains from refined grain products. Thus, a valid biomarker of whole grain intake would provide greater insight into the influence of diet on health and disease outcomes [31].

### Cane sugar and high fructose corn syrup

Carbon stable isotope abundance of ^13^C is a novel biomarker for cane sugar and high fructose corn syrup (HFCS). Cane sugar and HFCS are derived from C_4_ plants (includes molasses, brown and powdered cane sugar), making their intake measureable through ^13^C isotope measures [32]. Cook et al. [33] used ^13^C from blood glucose to determine its potential as a biomarker for cane sugar/HFCS; unfortunately, fasting glucose ^13^C levels were inadequate indicators of intake as gluconeogenesis caused ^13^C dilution. However, random plasma ^13^C measurements showed high correlations with consumption of cane sugar/HFCS from the previous meal (R^2^=0.90) [33] and serum ^13^C levels were shown to be correlated with SSB intake (r=0.18) in older adults [34]. Davy et al. [22] used fingerstick blood samples to measure the ^13^C isotope content, and reported higher correlations between SSB intake and ^13^C levels when compared to ^13^C venipuncture samples [34]. Correlations with added sugars (calories [kcals] and grams [g], r=0.37) and total SSB (kcal and g, r=0.35, 0.28) were noted [22]. Additionally, the reproducibility of ^13^C at 2 time points was found to be significant (r=0.87) [22].

Although moderate correlations were found for serum ^13^C to added sugars and SSB, there are limitations that require further research before the ^13^C isotope is considered a valid biomarker of cane sugar and HFCS. While high correlations were found for random plasma glucose ^13^C measures to cane sugar and HFCS, this only reflects extremely recent intake (i.e., the previous meal). For this reason as well as less invasiveness, fingerstick serum ^13^C measures may be a better choice (possibly reflects a longer intake period and is less invasive) for a cane sugar/HFCS intake biomarker; however, further research is warranted to determine the intake period reflected in the measurement.

Beet sugar and maple syrup, which only account for a small fraction of added sugars in the diet, are not captured by ^13^C measures as they are C_3_ plants, as well, honey is not included [32]. Thus, biomarkers for sugar intake that rely on ^13^C isotopes are only able to capture part of the general US population’s intake; even so, this does reflect a large portion of the consumed added sugars. Another limitation of using ^13^C isotopes is that corn is also a C_4_ plant; thus corn, corn derivatives and meat from animals that consumed corn are reflected in the measurement. ^13^C was shown to be correlated with whole corn intake and animal protein intake (r=0.15, 0.28, respectively) [34]. A second isotope, ^15^N, may be able to account and correct for animal protein intake [35]. Overall, ^13^C measures have shown promise as they can distinguish low from high sugar consumers [22], and have demonstrated significant correlations between SSB, added sugars and cane sugar/HFCS. Further research is needed to refine this added sugar biomarker and establish the intake period reflected by the measurement.

*Sugar*. Urinary sucrose and fructose have been investigated as possible biomarkers of sugar intake. Urinary sucrose, fructose and combined sucrose/fructose are associated with sugar intake (R^2^=0.86, 0.80, 0.89, respectively), and are reproducible (ICC=0.44, 0.81, 0.67, respectively) (examined using thirty 24-hour urine samples) [36]. Urinary sucrose and fructose concentrations did not significantly differ between normal and obese individuals when using a sugar controlled diet [37]. Kuhnle et al. [38] examined two analytical methods of determining urinary sucrose, gas chromatography mass spectrometry (GC-MS) and liquid chromatography mass spectrometry (LC-MS). GC-MS is able to identify more compounds than LC-MS, but the sample preparation for GC-MS is more labor-intensive and the analysis takes longer to conduct as it is examining more compounds than LC-MS.

Urinary sucrose and fructose are able to detect changes in sugar intake [36], classify an individual as a high or low sugar consumer and are suitable for those of normal and obese weight status [37]. As well, both the LC-MS and GC-MS analytic methods predicted urinary sucrose as a suitable biomarker of sugar intake [38]. However, a major limitation of urinary sucrose and fructose is the capability to only reflect short term intake. Further research is needed to develop a biomarker of total sugar intake that is reflective over a longer period of time (i.e., habitual intake).

### Whole grain wheat and Rye

Several studies have examined plasma alkylresorcinol (AR) concentrations as a possible whole grain wheat/rye biomarker. Total plasma AR increases with whole grain intake and decreases with refined bread intake after one week [39]. Fasting plasma AR demonstrated high to moderate reproducibility, ICC>0.88 (8 time points) [40], r=0.38 (2 time points) [41] and was correlated with whole grain intake (r=0.58) [31], (R^2^=0.94) [39], and rye/wheat intake (r=0.53) [41]. Non-fasting plasma AR concentrations were found to be significantly higher compared to fasting concentrations, however a moderate significant correlation of r=0.50 (p<0.05) between fasting and non-fasting was determined [41]. Red blood cell (RBC) AR increases and decreases with whole grain ingestion, and also correlates with plasma AR (R^2^=0.85) [39]. However, AR may be retained in RBC membranes during low AR intake [39]. Investigation of enterolactone (ENL), the main end-product of whole grains, revealed its poor function as a biomarker of whole grain intake, as it is a non-specific biomarker that has many dietary sources and varies greatly between genders [39]. AR homolog C17:0/C21:0 ratios have the potential to differentiate between types of whole grain intake, specifically wheat and rye [31,39,40].

3,5-dihydroxybenzoic acid (DHBA) and 3-(3,5-dihydroxyphenyl)-propanoic acid (DHPPA) are two metabolites of AR that are excreted through urine. Urinary DHBA and DHPPA were both found to be significantly correlated with total plasma AR (r=0.481, 0.450, respectively, no p values given) [42]. Additionally, cereal fiber intake was significantly correlated with DHBA and DHPPA, as well as plasma AR [42]. Recovery was shown to decrease with high AR doses; it could be that a 24-hour urine collection was not enough time to recover the high dose. DHBA and DHPPA were able to demonstrate a higher dose-response effect than plasma AR at low intake levels [43].

Total plasma AR appears to be a possible short term (half-life approximately four hours) biomarker of whole grain intake when assessing dose-response [43]; conversely, reproducibility has been shown over a 2-3 month period, which indicates a medium term marker. However, AR may accumulate over periods of high intake, thus over-estimating intake at high levels and under-estimating at low levels [31,40,43]. RBC AR may be a longer term indicator of whole grain intake than plasma AR, as they retain AR. Urinary DHBA and DHPPA may provide a comparable indicator of whole grain intake as plasma AR, while being minimally invasive [42]. Further research is needed to assess effects of various other whole grains on the AR homolog C17:0/C21:0 ratio, in addition to determining the time period being reflected.

### Fats

The current lack of a valid total fat dietary biomarker has hindered research targeting direct relationships of fat intake with cardiovascular disease, as dietary fat intake assessment has largely relied on subjective data [4,44]. The composition of fatty acid intake can be reflected in the measurements of blood cholesterol (e.g., LDL, HDL); however, the role of genetics must be acknowledged as having an effect on blood levels as well [14]. Nonetheless, actual intake of specific fatty acids (mono-unsaturated [MUFA], poly-unsaturated [PUFA] and saturated fatty acids [SFA]), which may be indicators of disease risk, is difficult to capture [4,45]. Additionally, intake of omega-3 fatty acids, eicosapentaenoic acid (EPA) and docosahexaenoic acid (DHA), have been linked with reduced cardiovascular disease risk; however, current methods of determining actual intake have proven to be costly and time-consuming [46]. Thus, research is needed to develop biomarkers that are cost-effective and able to detect dietary fat/fatty acid intake.

### Total fat

Dietary biomarkers that represent total fat intake have demonstrated conflicting results. Fatty acid RBC concentrations of MUFA, PUFA and SFA do not appear to be adequate biomarkers of total fat intake, especially SFA. Also, EPA, DHA and oleic acid may provide short term biomarkers of relative intake but not total fat intake [47]. King et al. investigated the possibility of using a combination of fatty acids to create a biomarker of total fat intake. Using three different biological samples (i.e., RBC, plasma phospholipids (PL) and cholesterol esters (CE)), to measure fatty acid status, three prediction models were produced that had high sensitivity and specificity (all >90%) in discerning between low fat/high fat intakes [44]. *Trans*-fats were a common indicator of total fat intake for all models, but it may be less useful as a biomarker as *trans*-fats are being removed from many foods. RBC markers may be a useful long term marker, as the RBC turnover is approximately 120 days; RBC also showed smaller changes in fatty acid composition compared to PL and CE measures [44]. Thus, utilizing a combination of various fatty acids may prove to be a biomarker of total fat intake.

### Fatty acids

Several studies examining biomarkers of relative fatty acid intakes have produced favorable outcomes. PUFA measured in adipose tissue showed strong correlations with dietary intake (r=0.15-0.58), specifically linoleic and alpha-linolenic acids [45]. Others have suggested that n-6 and n-3 PUFA in PL is a long-term biomarkers of relative intake (r=0.16, 0.29, respectively) [48]. A study comparing pre- to post-menopausal women reported a significant correlation between RBC and PUFA in post-menopausal women (r=0.39), but not pre-menopausal (r=0.17) [49], and correlations between RBC MUFA and relative intake (r=0.40-0.48) [49]. Also, plasma MUFA *cis*18:1n-9 was reported to be a long-term biomarker for total MUFA (r=0.22) [48]. RBC Oleic acid was found to be a valid biomarker of intake (r=0.45-0.47) [49], but RBC SFA does not appear to be a valid intake biomarker [49]. However, serum SFA 15:0 correlated with total SFA dietary intake (r=0.19) [48]. Adipose *trans*-fatty acids were also shown to correlate significantly with dietary intake (r=0.43) [45].

According to existing literature, adipose and plasma PUFA levels appear to be the best indicators of relative intake; RBC PUFA levels warrant additional research as correlations differed between population groups. RBC and plasma MUFA appear to be valid measures of MUFA intake, while RBC SFA does not appear to be a valid indicator of intake. Serum SFA measures show potential as biomarkers, but *trans*-fatty acid biomarkers may not be as useful due to reductions in the food supply in recent years.

### Essential fatty acids

Alpha-linolenic and linoleic acid are two essential fatty acids (EFA). Significant tissue-dietary correlations of alpha-linolenic and linoleic acid, respectively, in adipose tissue (r=0.51, 0.52), fasting blood (r=0.38, 0.43) and fasting plasma levels (r=0.39, 0.41) have been reported [50]. Others [49] have noted a significant correlation between RBC linoleic acid and relative dietary intake (r=0.23 pre-menopausal, 0.39 post-menopausal), but not for RBC alpha-linolenic acid. Fasting blood is comparable in results to plasma and adipose tissue, less expensive and less invasive than adipose tissue sampling. Thus, whole blood measures appear to be the ideal indicator of long-term linoleic acid intake, and possibly alpha-linolenic acid [50].

### Eicosapentaenoic acid and docosahexaenoic acid

EPA and DHA are omega-3 fatty acids primarily obtained from fish consumption [46]. Levels of plasma EPA and DHA, when compared to their relative dietary intake, produce significant correlations (EPA, r=0.57 males, 0.60 females; DHA, r=0.57 males, 0.30 females) [51]. The stable isotope ^15^N is associated with fish intake; thus, levels of EPA and DHA ^15^N were assessed in blood and hair samples. Dietary EPA and DHA were correlated with blood ^15^N levels (r=0.47, 0.46, respectively) [46]. Hair ^15^N was correlated with dietary EPA and DHA (r=0.83, 0.84, respectively) [52]. However, because ^15^N can be influenced by intake of other sources of animal protein (beef, etc.) feeding studies should be completed to further evaluate this biomarker’s specificity. As the availability of EPA and DHA fortified food items (bread, cereal, etc.) and supplements has increased over the past several years, and due to the amount of EPA and DHA varying within types of fish, this biomarker must be acknowledged to be a biomarker of EPA and DHA intake and not fish consumption.

Plasma EPA and DHA may be useful dietary biomarkers of their respective intake; further research is required to determine the time-period of intake reflected. Blood and hair ^15^N both provide accurate biomarkers of EPA and DHA intake. The turnover of EPA and DHA differ, thus RBC ^15^N levels may be providing indicators for two different time periods [46]. Hair ^15^N is able to reflect the previous two months of intake [52]. Plasma EPA and DHA, RBC and hair ^15^N all show potential as biomarkers of EPA and DHA intake; yet, further research is needed to determine dose-response as well as intake periods being measured.

An additional biomarker of omega-3 fatty acid intake, known as the Omega-3 Index, is the sum of EPA and DHA in RBC membranes, expressed as a percent of total fatty acids [53]. The validity and dose-response of the index was assessed by having 57 subjects randomized into 4 varying dose supplementation groups and was then compared to plasma phospholipid and whole blood EPA and DHA. Correlations between the Omega-3 Index and plasma phospholipid and whole blood EPA and DHA were both found to be significantly correlated, r=0.91, p<0.001, p<0.0001, respectively. Additionally, significant dose-responses were demonstrated between the varying intake levels. Significant changes from baseline levels were also found in all intake groups, with the exception of the 2 highest intake levels, which may indicate a ceiling effect [53]. Additional testing is required to assess the reliability of the Omega-3 Index, as well as the time period being assessed.

### Olive oil

Lower incidences of cardiovascular disease have been associated with diets where olive oil is a major contributor to fat intake [54]. Tyrosol and hydroxytyrosol are two phenolic compounds derived from olive oil intake. Tyrosol shows a strong dose-response effect in 24-hour urine samples, as well as similar recovery for a single dose and a week of sustained doses (16.9%, 19.4%, respectively). Hydroxytyrosol had a recovery of 78.5% after a single dose and 121.5% recovery after a week of sustained intake. This reveals that hydroxytyrosol probably accumulates as the recovery was higher than the intake of olive oil; additionally, hydroxytyrosol can also be derived from other sources, including endogenous sources [54]. Although further research is needed, tyrosol shows promise as a biomarker of olive oil intake.

A dietary biomarker of protein intake may be useful for determining nutritional status (over/under nourished). In addition, animal protein intake has been linked to increased risk of cancer, obesity, diabetes and the metabolic syndrome [55]. However, research determining the long-term effects of dietary protein intake is lacking due to the absence of a valid biomarker.

### Total protein

Urinary nitrogen is a valid method of assessing total protein intake, though several limitations exist. A comparison of a 28-day feeding study with multiple 24-hour urine nitrogen outputs produced a correlation of 0.99. When the time period is reduced to a single observation, the correlation is reduced to 0.50, but improves to a correlation of 0.95 with 18 days (p values not reported) [56]. To obtain the most accurate measurements, individuals should maintain a constant daily intake and be in nitrogen balance. Urinary nitrogen may underestimate high protein intake levels and overestimate at low intake levels, yet it is considered an adequate biomarker of protein intake. It is suggested that multiple 24-hour urine samples are needed to fully establish protein status [56].

### Animal protein

As discussed previously, isotopes ^13^C and ^15^N are potential dietary biomarkers for added sugars and fatty acids [22,32-35,46,52]. These isotopes have also been evaluated for their potential to measure animal protein intake via ^15^N and ^13^C hair, yet baseline measurements showed low correlations with dietary intake (R^2^=0.17, 0.44, respectively). A decrease in both isotopes with decreased protein intake has been reported, but not a significant increase with increased protein intake after four weeks. Thus, hair ^15^N and ^13^C do not appear to be valid short term dietary biomarkers of protein intake, but further research is needed to determine if they could be valid longer-term biomarkers [55]. Several potential biomarkers of red meat intake have been identified, creatinine, taurine, 1-methylhistidine, and 3-methylhistidine. These components are specific to meat intake and are excreted in the urine [57]. Two randomized crossover studies examined the mean levels of each component with four dietary conditions, three varying levels of red meat intake and one vegetarian diet. All components demonstrated a significant dose-response to the increase of red meat intake (P_trend_<0.0001). Furthermore, 1-methylhistidine and 3- methylhistidine demonstrated significant differences in the means across the four dietary conditions (all p<0.03). Taurine and creatinine did not appear to be as sensitive to intake and were not able to distinguish between the low red meat and the vegetarian diets. (p=0.95, 0.88, respectively) [57]. 3- methylhistidine and creatinine can be formed during muscle catabolism, thus, as markers of red meat intake they could potentially be falsely elevated. Furthermore, 3- methylhistidine was shown to have greater variance among participants on the same diet; the same was not demonstrated with 1- methylhistidine, which may be the most promising biomarker of the four components. This study had participants consume the respective diet for 15 days, with three 24-hour urine collections occurring on the final three days. Because no washout period was used, it is difficult to determine the time period measured. The half-lives of 1- and 3- methylhistidine are reported to be approximately 12 hours; thus, they are both considered short term biomarkers of red meat intake [57]. Further research reporting the reproducibility and the intake period being measured is needed.

### Various foods/dietary components

Table 2 presents a summary of the various food/dietary component biomarker studies that could not be categorized within a macronutrient category, as follows: caffeine, citrus, cocoa, garlic and wine.

**Table 2:** Summary of recent biomarker studies on various food/dietary components

**Food/Dietary component**	**Reference**	**Biomarker (Sample size)**	**Biological sample**	**Analytic procedure**^**a**^	**Biomarker class**^**b**^	**Validity**^**c,d**^**(p value)**	**Reproducibility**^**d**^**(p value)**	**Sensitivity**^**e**^
Caffeine	Crews et al.(2001)	Caffeine (137X) (8 adults)	Urine (24 hr)	HPLC	Short term			
		Caffeine Metabolite: 17X	Urine (24 hr)	HPLC	Short term	R2=0.58*		
		Caffeine Metabolite : 17U	Urine (24 hr)	HPLC	Short term	R2=0.87*		
		Caffeine Metabolite: 1X	Urine (24 hr)	HPLC	Short term	R2=0.78*		
		Caffeine Metabolite: AFMU	Urine (24 hr)	HPLC	Short term			
Citrus	Heinzmann et al. (2010)	Proline Betaine (8 adults)	Urine (24 hr)	1H NMR	Short term	R2=0.40 (<0.0001)		+
	Lloyd et al. (2011)	Proline betaine (23 adults)	Urine (fasting)	FT-ICR-MS	Short term?			
		Hesperidin	Urine (fasting)	FT-ICR-MS				
		Nariruin	Urine (fasting)	FT-ICR-MS				
Cocoa	Llorach et al. (2009)	Urinary metabolome (10 adults)	Urine	HPLC-q-TOF	Short term			
Garlic	Verhagen et al.(2001)	S-allyl-mercapturic acid (ALMA) (101 male adults)	Urine (24 hr)	GC-MS	Short term			
Wine	Zamora-Ros et al. (2006)	Total resveratrol metabolites (TRMs) (20 adults)	Urine (fasting)	LC-MS/MS	Short term			+
		TRMs	Plasma (fasting)	LC-MS/MS	Short term			
	Zamora-Ros et al. (2009)	Resveratrol metabolites (1,000 adults)	Urine (fasting)	LC-MS/MS	Short term	r=0.895 (p<0.001)		+
	Rotches-Ribalta et al. (2012)	Resveratrol metabolites (10 males)	Blood & Urine	LC-ESI-MS/MS	Short term			

^a^HPLC, High-performance liquid chromatography; 1H NMR, 1H Nuclear magnetic resonance spectroscopy; FT-ICR-MS, Linear trap quadrupole-Fourier-transform ion cyclotron resonance mass spectroscopy ultra; HPLC-q-TOF, High-performance liquid chromatography with time of flight mass spectrometry; GC-MS, Gas chromatography-mass spectrometry; LC-MS/MS, Liquid chromatography-tandem mass spectrometry; LC-ESI-MS/MS Liquid chromatography-electrospray ionization/multi-stage mass spectrometry.

^b^Short term: hours/days; Medium term: weeks/months; Long term: months/years.

^c^Results of comparsion of biomarkers to an appropriate dietary assessment method.

^d^Representative values from the literature.

^e^Biomarker is able to detect changes in intake over time or distinguish high from low consumers; + = sensitivity has been demonstrated.

*Significance not reported.

### Caffeine

Caffeine intake is difficult to assess via questionnaires and dietary recalls, as caffeine concentrations can vary greatly among different foods/beverages and may not be present in many nutritional software databases. However, due to the potentially harmful side effects of high caffeine intake, it may be important to develop acceptable intake levels and a biomarker that reflects consumption [58]. Caffeine (137X) is broken down into four known metabolites in the urine, 17X, 17U, 1X, AFMU. Caffeine, in its un-metabolized form, and AFMU are greatly influenced by inter-individual differences (e.g., genetic variability) and are not acceptable indicators of caffeine intake. Although 17X is minimally influenced by genetic variability and shows significant correlation with caffeine intake (R^2^=0.58), it requires more research before it is considered a valid biomarker of intake. 17U and 1X are both minimally influenced by inter-individual differences, show high correlations with intake (R^2^=0.87, 0.78, respectively) and may be acceptable biomarkers of caffeine consumption [58].

### Citrus

Total fruit and vegetable intake is difficult to objectively quantify due to most biomarkers measuring the effect of fruit and vegetables on health outcomes (e.g., reduction of oxidative biomarkers [59]) or intake of non-specific nutrients, such as Vitamin C (which is found/added in many dietary items) [4]. However, proline betaine was identified through nutrimetabolomic metabolic profiling as a possible marker of citrus consumption, which may be able to identify true intake of citrus fruits. Proline betaine was shown to be sensitive (86.3%), specific (90.6%) and significantly correlated with citrus consumption (R^2^=0.40). A limitation of proline betaine is its rapid urinary excretion, (i.e., 24 hours after intake) [21]. Lloyd et al. [60] demonstrated the ability of urinary proline betaine levels to differ among low, medium and high citrus consumers after an overnight fast. Levels of proline betaine were assessed throughout the study; however, oranges were the only citrus source used. Additionally, two metabolites, specific to orange intake, were identified: hesperidin and nariruin. However, they were found to be insufficient indicators of citrus intake. The study concluded that additional sensitivity and specificity of proline betaine, with additional intake of varying citrus foods, is necessary before it can be considered a useful dietary biomarker [60].

### Cocoa

Cocoa is a major source of phytochemicals (phenolic compounds), which have been shown to improve cardiovascular health and antioxidant status [61]. A study utilizing metabolomic metabolic profiling identified twenty-seven cocoa urinary metabolites that occurred over the 24-hour period following intake [61]. Additional research on the various identified cocoa metabolites should be conducted in order to develop a valid biomarker of cocoa intake.

### Garlic

It has been hypothesized that garlic may provide chemo-preventive effects; thus, the development of a biomarker of garlic intake may enhance research targeting cancer prevention, as well as prevention of other chronic disease [62]. S-allyl-mercapturic acid (ALMA) has been identified as a urinary metabolite of dietary garlic intake. In a research investigation, the presence of ALMA was detected in the majority of garlic consumers (fifteen out of sixteen), while only two control subjects out of fourteen had detectable levels of ALMA. Therefore, ALMA appears to differentiate garlic consumers from non-consumers. However, ALMA is a short term biomarker of garlic intake as the half-life is approximately six hours, and ALMA may increase with other sources; it is not specific to garlic intake [62].

### Wine

Resveratrol, a phenolic compound found in wine, has been shown to be negatively correlated with cardiovascular disease [63]. A biomarker for wine intake may prove to be useful, as individuals may not always accurately report alcoholic beverage consumption due to social undesirability (2). Metabolites of resveratrol have been discovered in urine and plasma, and total resveratrol metabolites (TRM) were analyzed to determine exposure and responsiveness of wine intake. Plasma TRM have an extremely short half-life of approximately two hours, and only reflect very recent intake. Urinary TRM, however, may differentiate between wine drinkers and non-drinkers with high sensitivity and specificity (73%, 93%, respectively). TRM also show a strong dose-response effect. A limitation of TRM is that it only reflects intake of regular consumers and may prove less useful in intermittent consumers of wine [63].

A later study demonstrated the ability of urinary resveratrol metabolites to be significantly correlated with wine intake (r=0.895, p<0.001) and also established sensitivity (93.3%) and specificity (92.1%) between consumers and non-consumers [64]. Further research is needed to be able to objectively classify consumers into groups based on their wine consumption levels [65]. Certain limitations exist, such as resveratrol is not specific to wine intake and can be found in grapes, peanuts, and berries; furthermore, the amount of resveratrol can vary between types of wine [64].

Rotches-Ribalta et al. [66] used mass spectrometry to identify resveratrol metabolites after ingestion of red wine and grape extract. Seventeen metabolites were identified, including *trans-* and *cis-*resveratrol and *trans-* and *cis-*piceid. Significant differences were found between red wine consumption and pharmaceutical ingestion of grape extract, which suggests further research is needed to assess resveratrol metabolism. Specifically, it has been shown that resveratrol has low bioavailability and a complex diet (especially fiber intake) may affect the concentrations of the metabolites. Colonic microflora also play a role in producing resveratrol’s metabolites, which is in need of further investigation as intra-individual microflora variability is high [66,67].

## Conclusions

Biomarkers of dietary exposure should be valid, reproducible, able to detect changes in intake over time and be suitable for the general population. Yet, many of the dietary biomarkers reviewed appeared inadequate at meeting all of the aforementioned criteria (see Tables 1,and 2). The majority of reviewed studies only examined the validity of a biomarker (twenty-two studies); four studies evaluated reproducibility and eight studies evaluated the biomarker’s ability to be sensitive to changes in respective dietary intake. The best biomarkers available show validity, reproducibility and sensitivity; this review identified two biomarkers that met all three criteria: combined urinary sucrose and fructose for a sugar biomarker (33) and total plasma alkylresorcinol for a whole grain biomarker (28, 37). Additionally, fingerstick ^13^C measurements demonstrated validity and reproducibility for cane sugar/HFCS intake (19), and urinary proline betaine demonstrated validity and sensitivity for citrus consumption (18).

There are multiple factors that warrant investigation before many of these biomarkers can be more widely utilized in nutrition and health research. Genetics, age, type of specimen, time of year, and confounding dietary sources play a pivotal role in the feasibility and validity of dietary biomarkers. This literature review indicated more research was needed for many macronutrient biomarkers, as well as novel indicators of specific foods/dietary components intake which could not be categorized within macronutrients. Furthermore, few biomarkers demonstrated cost-effectiveness and non-invasiveness (e.g., hair or fingerstick vs. venipuncture or adipose tissue). Emphasis should be placed on developing biomarkers using samples that are minimally invasive with a low subject burden (e.g., fasting). The practicality of the measure is also an important consideration, including the accessibility, collection, processing, storage and analysis of the specimen [2]. Common limitations to this body of literature include small sample sizes (less than 20 participants) [21,33,36,38-40,43,47,51,54-58,61,63,66] and lack of variability in participant’s gender [40,42,44,47-49,51,55,57,62,66], race [45,46,50-52], and age [33,34,44,45,50,55]. It should be taken into consideration that is a limited nonsystematic review in the emerging area of dietary biomarkers. Furthermore, no quality assessment of the included literature was conducted; therefore, discretion should be used when interpreting findings.

### Future directions

Biomarkers are needed to provide objective measures of nutrient status, which is a commonly cited limitation of subjective dietary assessment methods. However, some dietary intake methods use biomarkers to validate the data being collected. As noted by The Institute of Medicine, the need to expand upon dietary assessment methods is critical [10]. Biomarkers that will allow for the assessment of specific consumption of items which could be deemed socially undesirable, such as sugar-sweetened beverages or high fat/saturated fat foods, without confounds of human subjective nature need to be developed [13]. Future research pertaining to biomarkers should emphasize the development of biomarkers for evaluating adherence to national recommendations for specific food groups such as the U.S. 2010 Dietary Guidelines (e.g., whole grains, fruits and vegetables, low fat/fat free dairy products, added sugar) [29]. Future research should be directed at refining existing biomarkers by accounting for confounding factors, establishing new indicators of specific food intake and developing techniques that are cost-effective, noninvasive, rapid and accurate measures of nutritional status. The emerging field of metabolomics in human nutrition, as well as the development of valid FFQ and the continued expansion of food metabolome databases will permit the identification of specific dietary components in food, produce more valid biomarkers of exposure to certain foods and possibly advance nutritional science research which aims to evaluate diet and disease relationships.

## Competing interests

The authors do not have any financial conflicts of interest to disclose, and all authors have read and approved the final version of this manuscript. No portion of this work has been or is currently under consideration for publication elsewhere, and no portion of this manuscript has been published or posted on the Internet.

## Authors’ contributions

VH, AD, PS, JS, ES, and BD contributed to the conception and design of the review as well as drafting and revising the manuscript. VH performed the literature search. All authors read and approved the final manuscript.

## Authors’ information

VH is a post-doctoral associate, PE is a professor, ES is an associate professor, and BD is an associate professor, Department of Human Nutrition, Foods, and Exercise, and JS is an assistant professor, Department of Human Development and Center for Gerontology, and AD is a professor, Department of Civil and Environmental Engineering, all at the Virginia Polytechnic Institute and State University, Blacksburg.
